# Supplementary material for: Triangular lattice quantum dimer model with variable dimer density
Source: Nat Commun. 2022 Oct 2;13:5799. doi: 10.1038/s41467-022-33431-5 (PMC9527248; doi:10.1038/s41467-022-33431-5)
Supplement: Supplementary file 1 — Supplementary Information [file 41467_2022_33431_MOESM1_ESM.pdf]

Supplementary Information for
"Triangular lattice quantum dimer model with variable dimer density"

Zheng Yan,[1] Rhine Samajdar,[2] Yan-Cheng Wang,[3] Subir Sachdev,[2,4,*] and Zi Yang Meng[1,†]

[1]*Department of Physics and HKU-UCAS Joint Institute of Theoretical and Computational Physics, The University of Hong Kong, Pokfulam Road, Hong Kong SAR, China*

[2]*Department of Physics, Harvard University, Cambridge MA 02138, USA*

[3]*Beihang Hangzhou Innovation Institute Yuhang, Hangzhou 310023, China*

[4]*School of Natural Sciences, Institute for Advanced Study, Princeton, NJ 08540, USA*

Supplementary Note 1: Relation between the dimer spectra and vison-pair correlations.

In this section, we examine two dynamical correlation functions. The first one is the conventional dimer correlation: defining the dimer operator as $D_i = 1$ $(= 0)$ when there is a (no) dimer on the link i, the dimer correlation function is given by $C_d(r_{i,j}, \tau) = \sum_{i,j} \langle D_i(\tau) D_j(0) \rangle - \langle D_i \rangle^2$. Then, $C_d(\mathbf{q}, \tau)$ can be computed via a Fourier transformation, following which the excitation spectrum $C_d(\mathbf{q}, \omega)$ is obtained using stochastic analytic continuation (SAC).

The second quantity of interest is the correlation function of another differently defined "dimer", namely, the vison-pair correlation function. This "dimer" is the vison-convolution (VC) operator, which is defined as $D_i^{vc} = V_{i_1} V_{i_2} d_i$, where $d_i = \pm 1$ when there is no/one dimer on link i of the reference configuration (for the one-dimer-per-site case, we have to choose a reference configuration to fix the gauge; for the two-dimer-per-site case, $d_i = 1 \, \forall \, i$). The idea is that if two visons are close to each other, sharing the same link, then D_i^{vc} on the link i can be represented as the product of these two vison operators, with i_1 and i_2 being the triangular plaquettes closest to the link i. Assuming the interaction of the visons is weak, this correlation function $C_d^{vc}(r_{i,j}, \tau) = \langle D_i^{vc}(0) D_j^{vc}(\tau) \rangle - \langle D_i^{vc}(0) \rangle^2 = \langle V_{i_1}(0) V_{i_2}(0) d_i V_{j_1}(\tau) V_{j_2}(\tau) d_j \rangle - \langle V_{i_1}(0) V_{i_2}(0) \rangle^2$ can be computed using Wick's theorem as the convolution of two vison operators,

$$C_d^{vc}(r_{i,j}, \tau) = \langle V_{i_1}(0) V_{j_1}(\tau) \rangle \langle V_{i_2}(0) V_{j_2}(\tau) \rangle d_i d_j + \langle V_{i_1}(0) V_{j_2}(\tau) \rangle \langle V_{i_2}(0) V_{j_1}(\tau) \rangle d_i d_j. \quad (1)$$

Here, d_i is constant for link i under the choice of a gauge, and can be taken outside the brackets.

Comparing the two abovementioned correlation functions, we see that one dimer can thus be treated as a vison pair deep in the quantum spin liquid (QSL) phase if the interaction effects among the visons are sufficiently weak. Further details in this regard can be found in the discussion accompanying Eq. (2) of Supplementary Ref. 1.

Supplementary Note 2: Phase diagram at $h = 0$.

Besides the phase diagram with $h = 0.4$ presented in the main text, we also study the phase diagram at $h = 0$ described by the Hamiltonian,

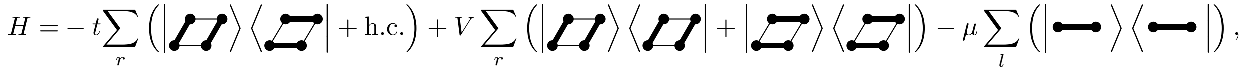

(2)

where the sum on r runs over all plaquettes (rhombi) including the three possible orientations. The kinetic term t, the potential term V, and the chemical potential μ are the same as in Eq.(1) of the main text; we set $t = 1$ as the unit of energy. As in the finite-h case, we impose a soft constraint requiring either one or two nearest-neighbor dimer(s) per site.

Since the Hamiltonian cannot flip single dimers on a bond in the absence of an h term, the dimer filling becomes a conserved quantity. First, we note that with $V = 1$ but varying μ, the system is described by the Rokhsar-Kivelson (RK) wavefunction of an equal superposition of dimer coverings [2], thus forming a QSL ground state. Moreover, the ground-state energy of the Hamiltonian [Supplementary Eq. (2)] at the RK point, without the μ term, is identically zero for a fixed filling ρ. A nonzero μ trivially makes the ground state favor one (two) dimers per site when $\mu < 0$ (> 0). Similarly, the 1/6- and 1/3-filling staggered phases are degenerate when $\mu = 0$. Hence, the (first-order) phase transition line separating these two staggered phases remains at $\mu = 0$.

---

* sachdev@g.harvard.edu

† zymeng@hku.hk

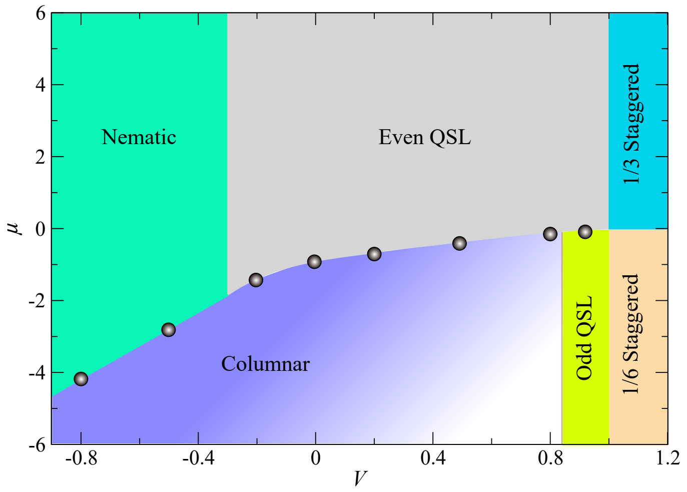

Supplementary Figure 1. Phase diagram of $h = 0$ case. The phase diagram, spanned by the V and μ axes, obtained from QMC simulations at $h = 0$. The QSL–nematic and QSL–columnar transitions are continuous, and the QSL–staggered transition is first-order. In the limit of exactly one dimer per site, a $\sqrt{12} \times \sqrt{12}$ valence bond solid (VBS) phase is known to exist between the odd QSL and the columnar phase. However, it is nearly degenerate with the columnar phase over a large region in our simulations—especially for larger sizes—and we depict this schematically by using a lighter shading for the columnar phase near the odd QSL.

For the phase boundaries determined by fixing μ and varying V, if the phases have the same filling, the chemical potential μ will not change their energy difference but just impart equal energy shifts. Therefore, in the vertical direction, the phase boundaries are just straight lines. Likewise, the horizontal phase boundaries, which separate phases of two different fillings, are also straight. Together, they give rise to the entire phase diagram in Supplementary Fig. **1**.

In the limit of large, negative V, the kinetic term becomes irrelevant. The Hamiltonian then becomes a classical one with competing terms set by μ and V. It is not hard to see that the phase transition line between the columnar and nematic phases is simply given by $V = \mu/3$ in this classical limit.

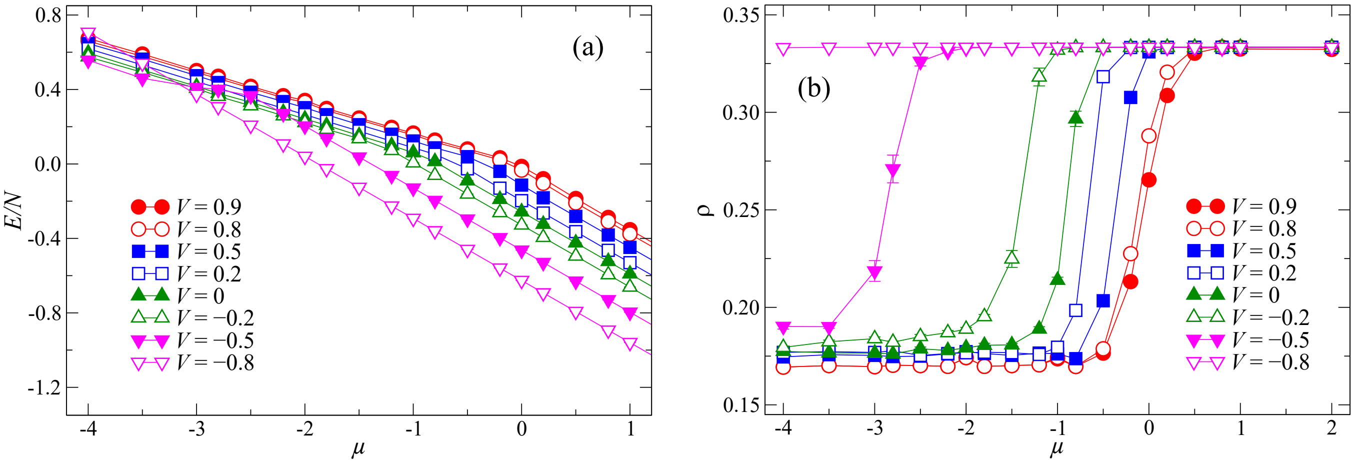

Supplementary Figure 2. The relation of Energy/filling and μ of different V. (a) Energy density E/N, and (b) dimer filling ρ for different V while scanning μ at a fixed size $L = 12$. The phase transition line progressively shifts towards more negative values of μ as V is decreased.

To understand the phase diagram at a quantitative level, we simulate the model at a fixed system size of $L = 12$. Clear first-order phase transitions arise between the columnar and nematic, the columnar and even QSL, and the odd and even QSL phases, as can be seen from the energy density E and the dimer filling ρ (Supplementary Fig. **2**). We have also studied the system-size dependence of the odd and even QSL transition at $V = 0.9$ with $L = 12, 18$ and

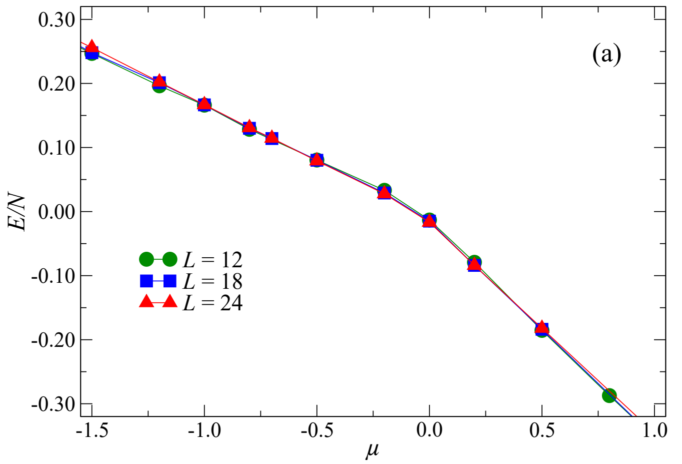

Supplementary Figure 3. **The relation of Energy/filling and μ of different L at $V = 0.9$.** (a) The energy densities for different system sizes exhibit similar behaviours as a function of μ. (b) The dimer filling ρ becomes sharper while the size increases. This data confirms an obvious first-order phase transition between the odd and even QSLs.

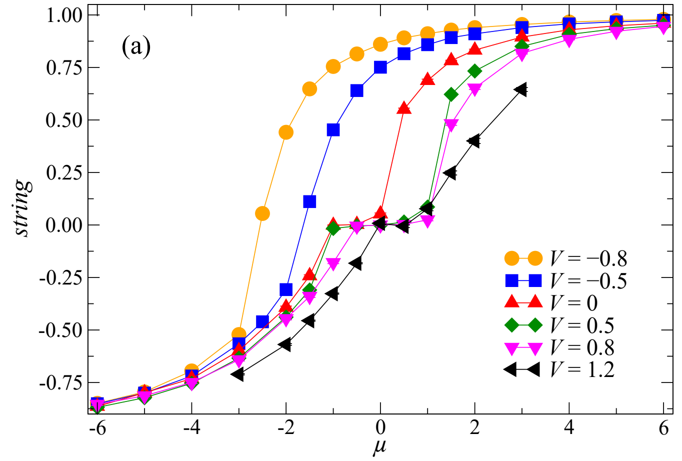

Supplementary Figure 4. **The relation of string/filling and μ of different V.** (a) The string operator $\langle string \rangle$ and (b) the dimer filling ρ as a function of μ for different values of $h = 0.4$, and $V = 0.8$, 0.5, 0, -0.5, -0.8 in a system of size $L = 16$. The system size simulated for $V = 1.2$ is chosen to be $L = 12$ due to the unit cell of the staggered order.

24. As shown in Supplementary Fig. **3**, the first-order phase transition also becomes more obvious as the system size increases, and there is no PM phase between the two QSLs unlike for the finite-h cases, which we now turn to discuss.

Supplementary Note 3: Additional data for the phase diagram at $h = 0.4$.

In this section, we provide the detailed data used to construct the phase diagram sketched in Fig. 1 of the main text.

First, the string operator $\langle string \rangle$ and the dimer filling ρ are used to distinguish between the PM and other phases. In Supplementary Fig. **4**, we present these observables as a function of μ for fixed $V = 0.8, 0.5, 0, -0.5, -0.8$. They clearly show the vanishing of the PM region as V is varied from positive to negative values. At $V = -0.8$, a clear first-order transition between the nematic phase (with $\rho \sim 1/3$) and the columnar phase (with $\rho \sim 1/6$) is manifest.

Moreover, starting from $h = 0$, we found that the extent of the PM phase increases with increasing h. To demonstrate this behavior, we measure the string operator at h =0.1, 0.2, 0.4 for a fixed V =0.9 and varying μ. The results are shown in Supplementary Fig. **5**. It is clear that at $h = 0.1$, $\langle string \rangle$ jumps from -1 to 1 at $\mu \sim 0$, close to the odd QSL to even QSL first-order transition observed at $h = 0$ for the phase diagram of Supplementary Fig. **1** in the previous section. However, as h increases, to 0.2 and 0.4, the intermediate PM phase, with $\langle string \rangle = 0$, separating the two QSLs becomes clearer.

Going back to the $h = 0.4$ phase diagram, on the positive (negative) μ side, one finds the 1/3 (1/6) staggered phase

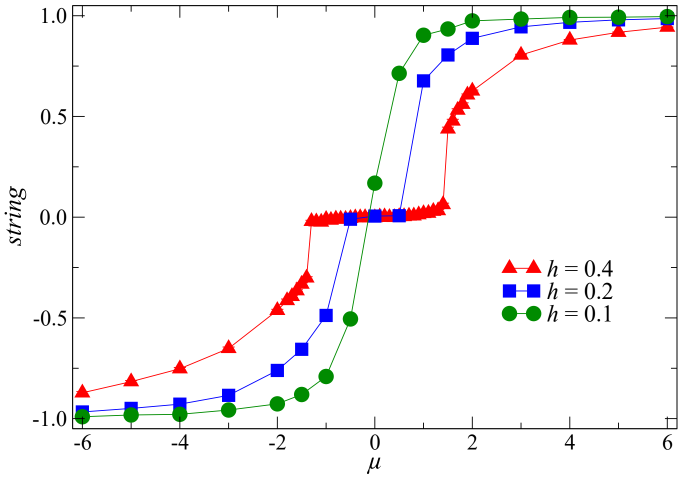

Supplementary Figure 5. **The relation of string and μ of different h.** The string operator as function of μ for $h = 0.1$, 0.2, 0.4, with $V = 0.9$ and a system size of $L = 16$. It shows the region of PM becomes larger when the h increasing.

and the even (odd) QSL near $V = 1$. As V approaches negative values, the phase transition between the QSL and the valence bond solid (VBS) phase is proposed to be continuous and in the O(3)* (O(4)*) universality class [1, 3] with large anomalous dimension exponents [4, 5]. However, we note that the precise nature of this topological transition is still largely unknown; here, we use the energy differences between different sectors to roughly estimate the position of the phase transition, following previous examples [6]. The basic idea is that the VBS state belongs to the (0,0) sector of dimer coverings on the torus geometry, and the other sectors—such as (1,1) with two topological defects

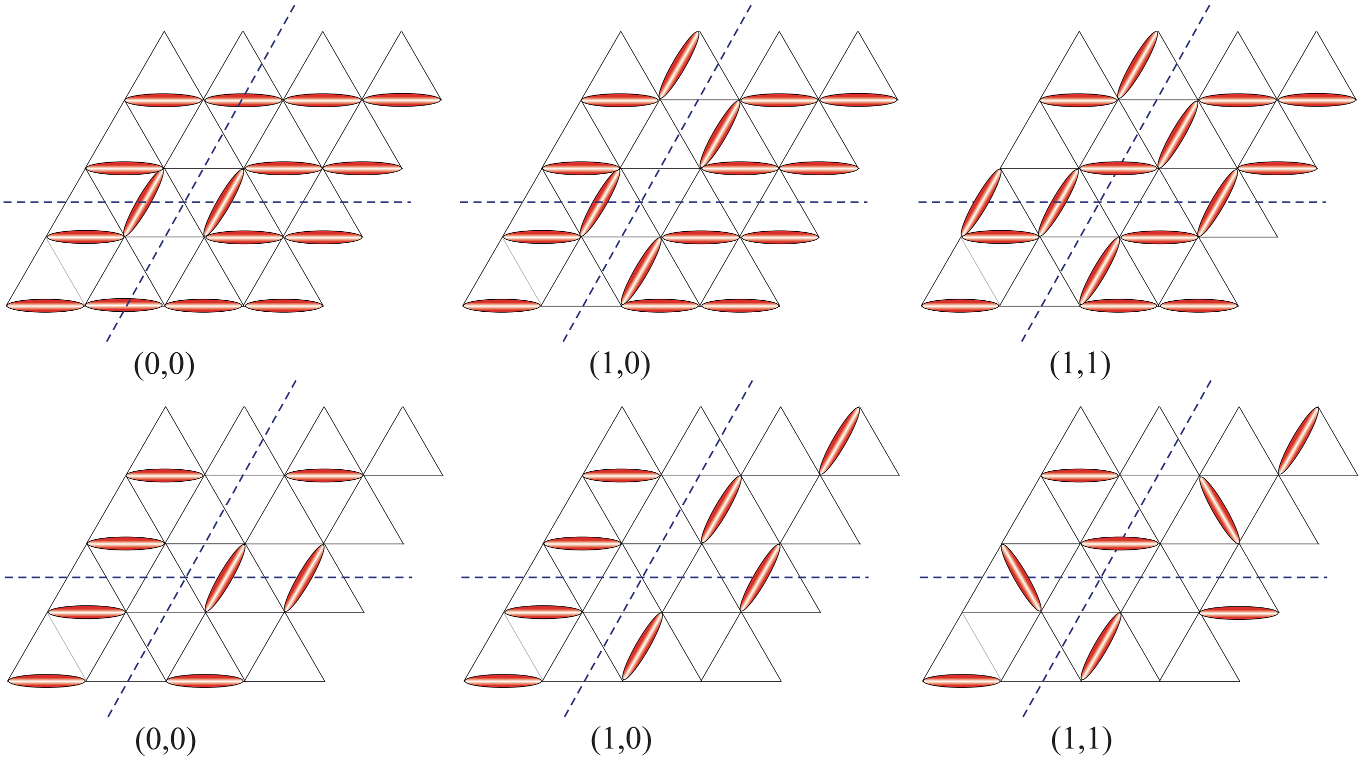

Supplementary Figure 6. **The definition of winding number.** The winding number is well-defined on a lattice with periodic boundary conditions. The number of dimers cut (modulo 2) along the two directions, as shown by the dashed lines, yields the winding number (x, y). The upper (lower) row arrays a few examples of dimer configurations with different winding numbers in the 1- (2)-dimer-per-site limit.

along the x, y axes—will cost more energy [7–10] as they create global domain walls. On the other hand, these sectors are topologically degenerate in the $\mathbb{Z}_2$ QSL phase. In the QMC simulation, we can prepare the dimer states in these different sectors, as shown in Supplementary Fig. **6**, and monitor their energy difference as we scan through the transition from the VBS to the $\mathbb{Z}_2$ QSL phase. As the system size increases, the energy difference vanishes inside the QSL and saturates to a finite value (note that the difference is scaled such that it is intensive) inside the VBS phase. The boundary between these two behaviors is then taken to be the boundary of the two phases.

The results of such an analysis are shown in Supplementary Fig. **7**, where we scan V for two different chemical potentials, $\mu = 3$ and $\mu = 6$. As the linear system size increases from $L = 12$ to 16, the energy difference (E/N on the y axis) between the topological sectors $(0, 0)$ and $(1, 1)$ remains nonzero in the nematic phase but becomes vanishingly small in the $\mathbb{Z}_2$ QSL phase. We use the vertical dashed lines to denote the transition points determined in this fashion, and these are the phase boundaries presented in the phase diagram of the main text.

Lastly, we discuss the phase transition between the 1/3- or 1/6-staggered VBS phases at large positive V and the corresponding QSL phases. In the phase diagram without h (Supplementary Fig. **1**), the staggered phases at both 1/3 and 1/6 fillings are separated from their proximate QSLs by first-order phase transitions. This behavior persists in the case of $h = 0.4$ as well, as illustrated by Supplementary Fig. **8**. Here, we plot the energy densities with the initial configuration chosen to be either the QSL or the staggered state, and find that the two different energy curves cross each other at the first-order transition point ~ 0.98 for two values of μ. The phase boundaries in the main text

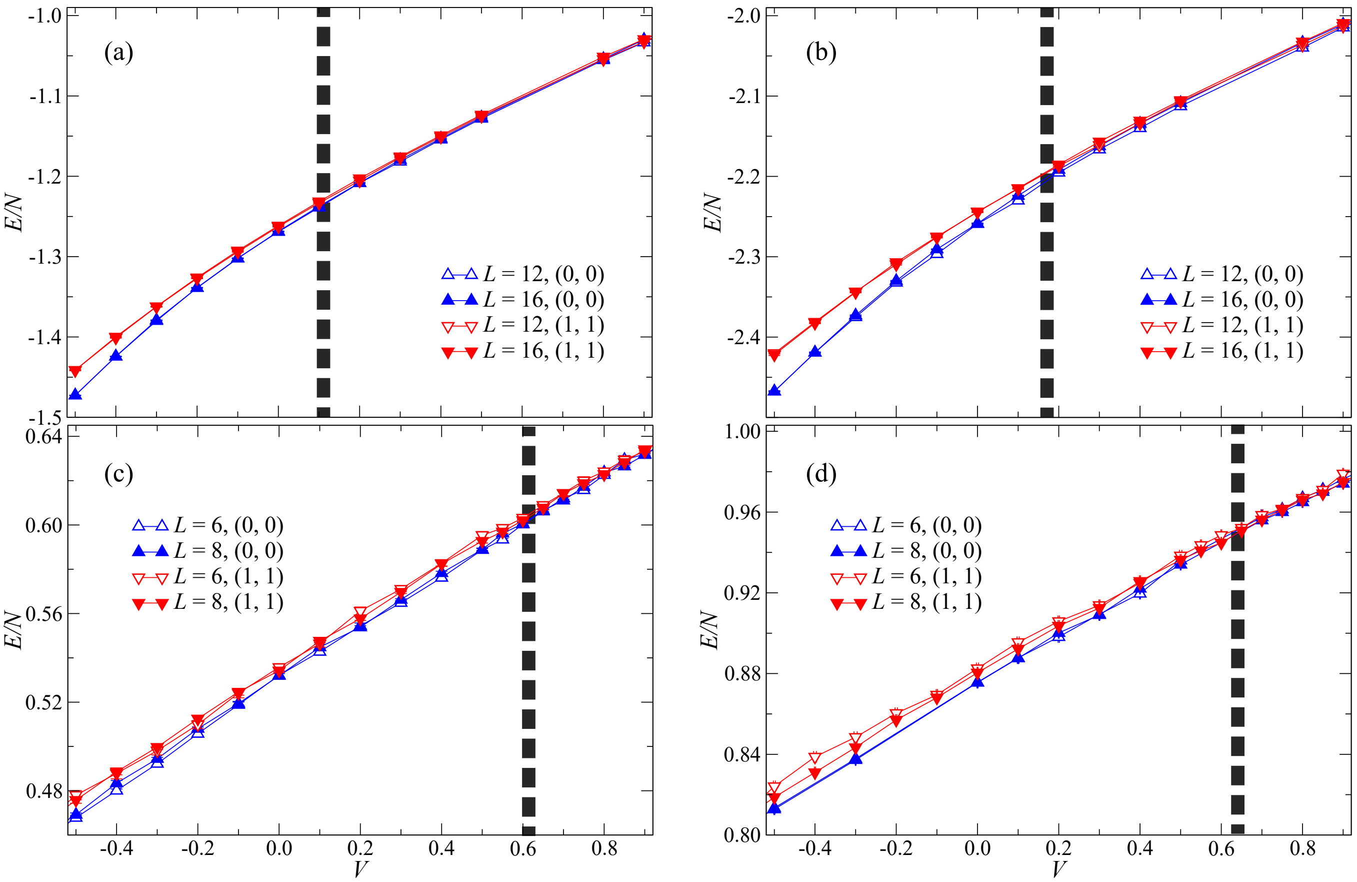

Supplementary Figure 7. **The energy difference of topological sectors.** The energy difference per site in different topological sectors as we scan V through the nematic VBS to even $\mathbb{Z}_2$ QSL phase transition for $\mu = 3$ (a), and $\mu = 6$ (b). The two sectors $(0, 0)$ and $(1, 1)$ have a finite energy difference in the nematic VBS and become degenerate in the QSL, especially as the system size increases. The vertical dashed lines denote the transition point within our simulation resolution. A similar approach is used to distinguish the columnar VBS and the odd QSL roughly at $\mu = -4$ (c), and $\mu = -6$ (d).

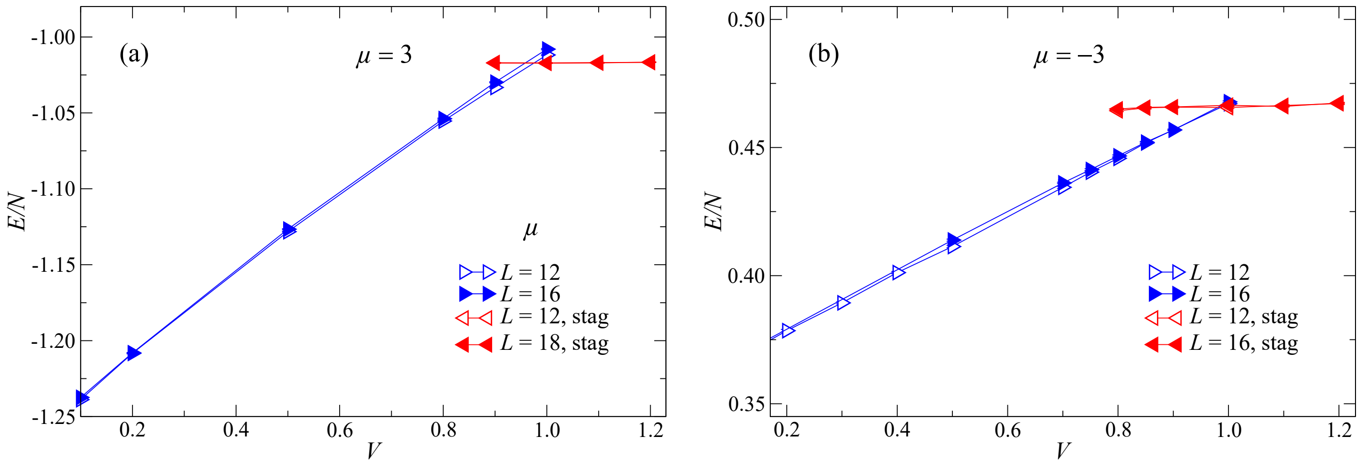

Supplementary Figure 8. **Energy cross for the first order phase transition.** The staggered phases belongs to different topological sectors than the QSL phases. The staggered-to-QSL phase transitions are found to be first-order by examining the energy density curves at (a) $\mu = 3$ and (b) $\mu = -3$. Clearly, the lines for the staggered and QSL phases cross at ~ 0.98, which is close to the $V = 1$ QSL–staggered phase transition point of the original dimer models (cf. Supplementary Fig. **1**).

are determined by this procedure.

---

[1] Z. Yan, Y.-C. Wang, N. Ma, Y. Qi, and Z. Y. Meng, Topological phase transition and single/multi anyon dynamics of Z_2 spin liquid, npj Quantum Mater. **6**, 39 (2021).
[2] D. S. Rokhsar and S. A. Kivelson, Superconductivity and the Quantum Hard-Core Dimer Gas, Phys. Rev. Lett. **61**, 2376 (1988).
[3] K. Roychowdhury, S. Bhattacharjee, and F. Pollmann, $\mathbb{Z}_2$ topological liquid of hard-core bosons on a kagome lattice at 1/3 filling, Phys. Rev. B **92**, 075141 (2015).
[4] S. V. Isakov, R. G. Melko, and M. B. Hastings, Universal Signatures of Fractionalized Quantum Critical Points, Science **335**, 193 (2012).
[5] Y.-C. Wang, X.-F. Zhang, F. Pollmann, M. Cheng, and Z. Y. Meng, Quantum Spin Liquid with Even Ising Gauge Field Structure on Kagome Lattice, Phys. Rev. Lett. **121**, 057202 (2018).
[6] A. Ralko, M. Ferrero, F. Becca, D. Ivanov, and F. Mila, Zero-temperature properties of the quantum dimer model on the triangular lattice, Phys. Rev. B **71**, 224109 (2005).
[7] Z. Yan, Global scheme of sweeping cluster algorithm to sample among topological sectors, Phys. Rev. B **105**, 184432 (2022).
[8] Z. Zhou, C. Liu, Z. Yan, Y. Chen, and X.-F. Zhang, Quantum dynamics of topological strings in a frustrated Ising antiferromagnet, npj Quantum Mater. **7**, 1 (2022).
[9] Z. Zhou, D.-X. Liu, Z. Yan, Y. Chen, and X.-F. Zhang, Quantum tricriticality of incommensurate phase induced by quantum domain walls in frustrated Ising magnetism, arXiv preprint arXiv:2005.11133 (2020).
[10] Z. Yan, Z. Zhou, Y.-C. Wang, Z. Y. Meng, and X.-F. Zhang, Sweeping quantum annealing algorithm for constrained optimization problems, arXiv preprint arXiv:2105.07134 (2021).
